# Can a Glove-Coach Technology Significantly Increase the Efficacy of Cardiopulmonary Resuscitation on Non-healthcare Professionals? A Controlled Trial

**DOI:** 10.3389/fcvm.2021.685988

**Published:** 2021-12-09

**Authors:** Michele Musiari, Andrea Saporito, Samuele Ceruti, Maira Biggiogero, Martina Iattoni, Andrea Glotta, Laura Cantini, Xavier Capdevila, Tiziano Cassina

**Affiliations:** ^1^Department of Anaesthesiology, Fribourg Cantonal Hospital (HFR), Villars-sur-Glâne, Switzerland; ^2^University Clinic for Anesthesiology and Pain Therapy Inselspital, Bern University Hospital, Bern, Switzerland; ^3^Department of Anaesthesiology, Bellinzona Regional Hospital, Bellinzona, Switzerland; ^4^Department of Intensive Care Unit, Clinica Luganese Moncucco, Lugano, Switzerland; ^5^Clinical Research Unit, Clinica Luganese Moncucco, Lugano, Switzerland; ^6^Department of Internal Medicine, Clinica Luganese Moncucco, Lugano, Switzerland; ^7^Montpellier University Hospital, Department of Anaesthesia and Intensive Care, Montpellier, France; ^8^Cardiocentro Clinic, Intensive Care, Lugano, Switzerland

**Keywords:** non-healthcare professional, wearable technology, cardiopulmonary resusciation, technology innovation, cardiac arrest

## Abstract

**Introduction:** Cardiovascular accidents are the world's leading cause of death. A good quality cardiopulmonary resuscitation (CPR) can reduce cardiac arrest-associated mortality. This study aims to test the coaching system of a wearable glove, providing instructions during out-of-hospital CPR.

**Materials and Methods:** We performed a single-blind, controlled trial to test non-healthcare professionals during a simulated CPR performed on an electronic mannequin. The no-glove group was the control. The primary outcome was to compare the accuracy of depth and frequency of two simulated CPR sessions. Secondary outcomes were to compare the decay of CPR performance and the percentage of the duration of accurate CPR.

**Results:** About 130 volunteers were allocated to 1:1 ratio in both groups; mean age was 36 ± 15 years (min–max 21–64) and 62 (48%) were men; 600 chest compressions were performed, and 571 chest compressions were analyzed. The mean frequency in the glove group was 117.67 vs. 103.02 rpm in the control group (*p* < 0.001). The appropriate rate cycle was 92.4% in the glove group vs. 71% in the control group, with a difference of 21.4% (*p* < 0.001). Mean compression depth in the glove group was 52.11 vs. 55.17 mm in the control group (*p* < 0.001). A mean reduction of compression depth over time of 5.3 mm/min was observed in the control group vs. 0.83 mm/min of reduction in the glove group.

**Conclusion:** Visual and acoustic feedbacks provided through the utilization of the glove's coaching system were useful for non-healthcare professionals' CPR performance.

## Introduction

Cardiovascular accidents are the world's leading cause of death (1), with a constant increase in cardiac arrest incidence in the out-of-hospital setting, reaching 140 cases per 1,00,000 subjects (2). The quality of cardiopulmonary resuscitation (CPR) has been reaffirmed by the American Heart Association (AHA) consensus statement, as “the primary component in influencing survival from cardiac arrest” (3). With the aim to provide early treatment in patients with cardiac arrest, basic life support (BLS) techniques have been taught for many years to an increasing number of laypersons worldwide. Since the initial positive effect obtained after the BLS introduction, the survival rate has not changed significantly during the years, still remaining <11% (2). To date, the reasons behind these data are still not clear.

Survival after cardiac arrest is directly related to the effectiveness of CPR, which must ensure adequate myocardial oxygen delivery (4, 5) through adequate coronary perfusion pressure. This is generated by the difference between aortic and right atrium diastolic pressures during the relaxation phase of chest compressions (6, 7). Chest compression rate and depth are the two main CPR determinants of coronary perfusion pressure (3). In particular, chest compressions should have a rate of 100–120 per min and a depth of at least 50 mm in adult patients (8). It has been shown that, even if performed by trained paramedics, chest compressions are often inadequate (9–11), with a potential negative impact on CPR outcomes (12, 13). It is thus likely that BLS performed in the out-of-hospital setting by laypersons could result in an inadequate myocardial blood flow, with a consequent negative impact on patients' survival rate.

During a prolonged cardiac arrest, mechanical chest compressions invariably degrade over time (14), mainly because rescuers do not perceive fatigue, which has a negative impact on their performance (15, 16). Moreover, a large interindividual variability has been described in chest compression rate since the beginning of CPR even among trained BLS providers (12). The CPR Quality Summit Investigators, the AHA Emergency Cardiovascular Care Committee, and the Council on Cardiopulmonary, Critical Care, Perioperative, and Resuscitation in the AHA consensus statement have specifically defined the quality of CPR as a major public health problem (3).

Systems that provide quantitative feedback of quality parameters during CPR have been shown to potentially improve the quality of CPR (17, 18), but they have not been conceived to assist laypersons during this procedure. As clearly affirmed by the AHA consensus statement: “Although some software (automated algorithms) and hardware solutions currently exist (smart backboard, dual accelerometers, reference markers, and others), continued development of optimal and widely available CPR monitoring is a key component to improved performance” (3).

The aim of this study was to test the methodologies applied to a new wearable device, expressing the concept of a coaching system potentially applicable to laypersons and involving vocal and visual instructions during out-of-hospital CPR, and also a real-time vocal and visual feedback during the cardiac massage. This prevents degradation of chest compression mechanic quality over time, thus increasing the overall quality and effectiveness of CPR.

## Materials and Methods

We performed a blinded, parallel group, controlled trial to test the performance of adult non-healthcare professionals' volunteers during a simulated CPR in a primary reference center for cardiology in Lugano (CardioCentro, Switzerland), from March 6 to April 26, 2019. As the first practical application of the device, for safety reasons and before applying it *in vivo*, the electromechanical model AmbuMan (Ballerup, Denmark) (19, 20) was used to perform and record CPRs. Volunteers were recruited from a midwestern community in Lugano, with a random selection from a large pool of students, teachers, acquaintances, excluding all doctors, nurses, and paramedics with a previous ACLS formation.

### The Glove Coaching System

**Figure 1:**
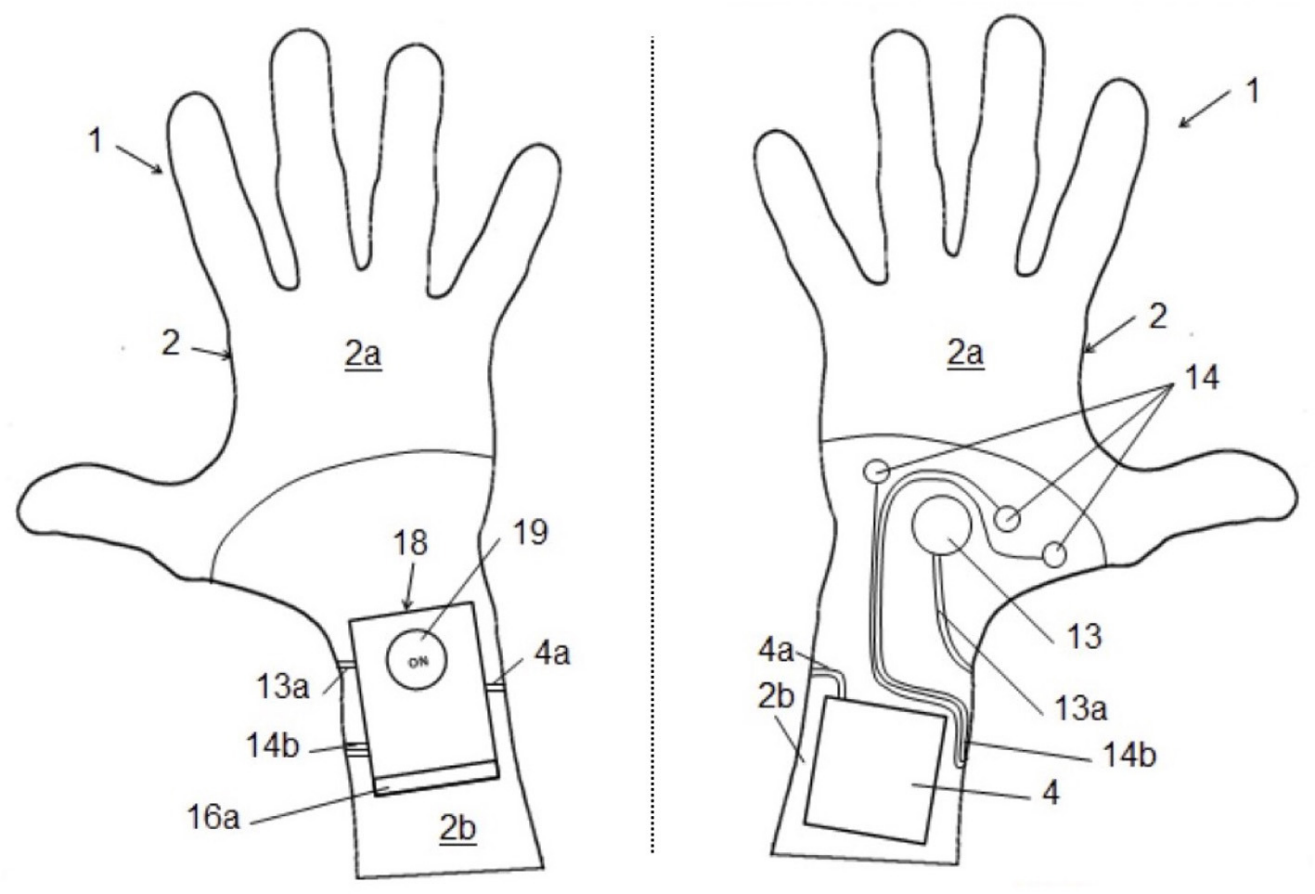
Glove-specific functioning: glove prototype from the patent scheme. The numbers are referred to the details discussed in Supplementary Material.

Our research group developed a new prototype wearable device conceived to improve CPR accuracy, a glove able to guide rescuer manipulations through visual and vocal feedbacks (Patent Swiss number 00112/15, Lugano, Switzerland, patent European number MI2015A000132, Milano, Italy). The glove was equipped with a microprocessor (Figure 1) that communicated with a personal computer, which is able to encode the received signal, providing vocal, and visual feedback during the CPR procedure. The decision to pass the signal through a computer was intended only for this prototype, as the aim was to check the coaching system and not the prototype itself. During the CPR, through an embedded microprocessor, the glove detected depth and frequency applied on the patient's chest. A specific algorithm glove-developed measured frequency and amplitude of each compression through vocal and visual warnings and guided the rescuer throughout the entire CPR procedure, acting as a coach.

### Volunteers

Volunteers were recruited 1 month before the start of the study and alternatively allocated into two groups; information such as age, sex, and previous experience of BLS was recorded. The glove group was considered as the test group, wearing and using the glove; the second group without glove was intended as a control group, performing CPR as standard for non-healthcare professionals, without any supported coaching system. According to the standard 2015 ACLS protocol (3), volunteers of both groups were asked to perform five cycles of 30 chest compressions paused by two respiratory inflation for about 10 min of CPR. A short explanation and a demonstration of the CPR were provided before the study data collection. All data collected were electronically registered during the CPR simulation.

### Data Registration

**Figure 2:**
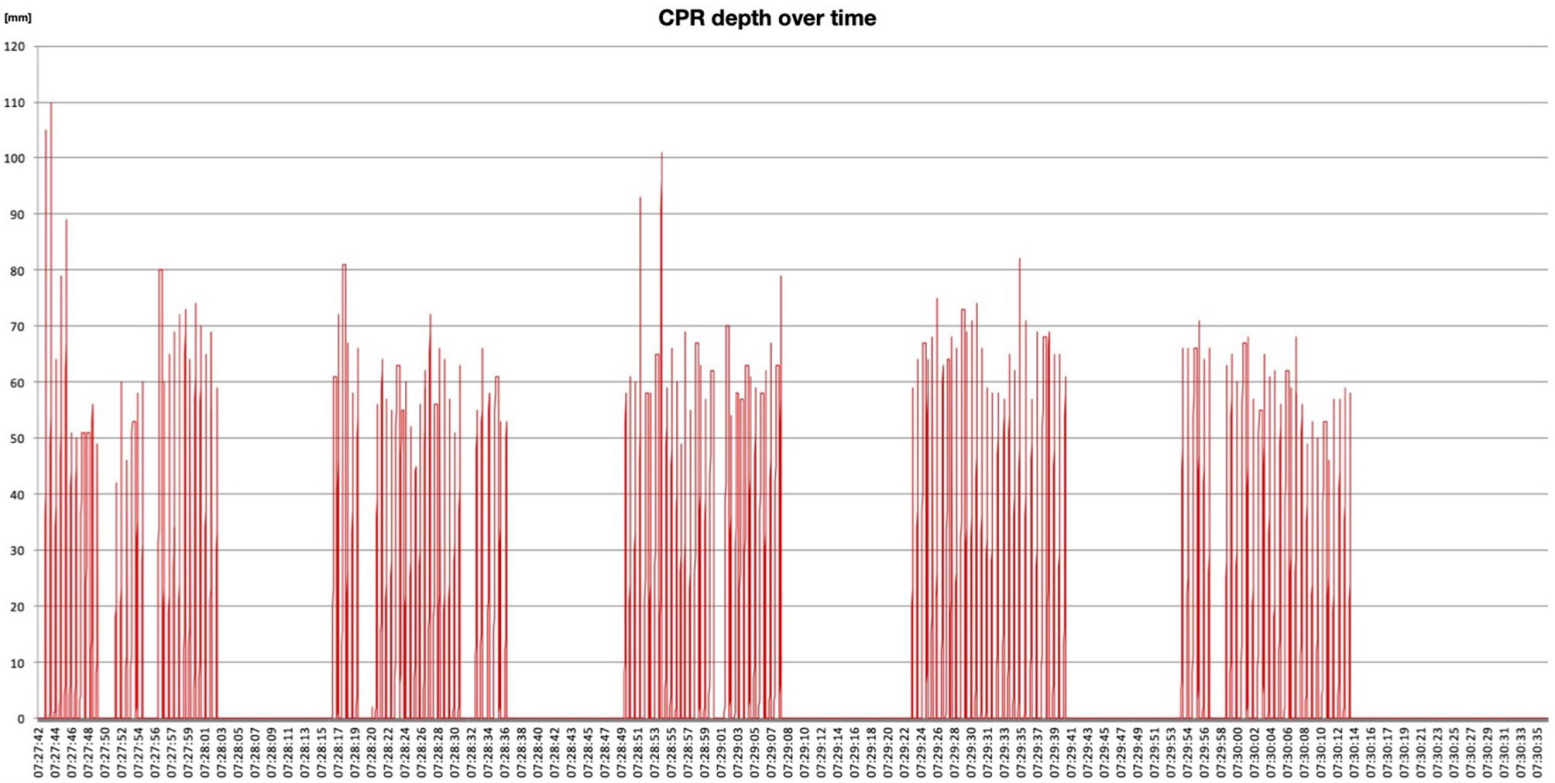
CPR plan: example of one registration during CPR simulation, reporting chest compression frequency and depth over time.

In both groups, a computer program enclosed to Ambuman mannequin constantly measured the compression amplitude range; normal values were established between 5 and 6 cm. Similarly, CPR frequency was regularly measured and considered adequate by the electronic system within an interval of 90–120 compression/min. This program registered all information, without returning any feedback about compression depth and rate, neither to investigators nor to the participant; it drew a depth-frequency curve over time (Figure 2). Each volunteer's CPR performance was thus considered as blinded, allowing at the same time to register all data about CPR.

### Outcome Measures

The primary outcome was to compare the accuracy of simulated CPR sessions performed by lay volunteers of both groups, in terms of chest compression frequency and depth. Secondary outcomes were to report decay of performance between two groups regarding chest compression depth evolution over time and the percentage of time in which the candidate performed accurate CPR.

### Statistical Analysis

Following a pilot study on a sample of 100 measurements, a compression power analysis was conducted: assuming an anticipated combined rate of accuracy in both depth and frequency of chest compressions of 83% in the control group and 91% in the glove group, an alpha error of 0.05 and a power of 80%, a total of 276 compressions per arm were needed. We assumed a difference of 10% in the mean accuracy of chest compression with regard to each of the two parameters studied to be clinically significant. Sixty-five volunteers per arm were thus recruited to compensate for dropouts due to possible technical failures of the prototype and asked to deliver five cycles of compressions each. Descriptive statistic was performed to summarize the collected clinical data. Data were presented as mean (SD) or median (IQR) for continuous variables, according to data distribution, and as absolute number (and percentage) for categorical variables; data distribution was verified by Kolmogorov–Smirnov test. A one-way repeated measures ANOVA test was used to detect differences in the two groups (95% CI). The statistical analysis was conducted with SPSS software. The investigator performing the statistical analysis was blinded with regard to the allocation of the volunteers. Data are expressed as mean and standard deviation (SD). A *p*-value of <0.01 was assumed as statistically significant.

### Ethics Committee Permission

The Ethics Committee approved the protocol, without requiring that the informed consent form as an electronic model is used.

## Results

**Figure 3:**
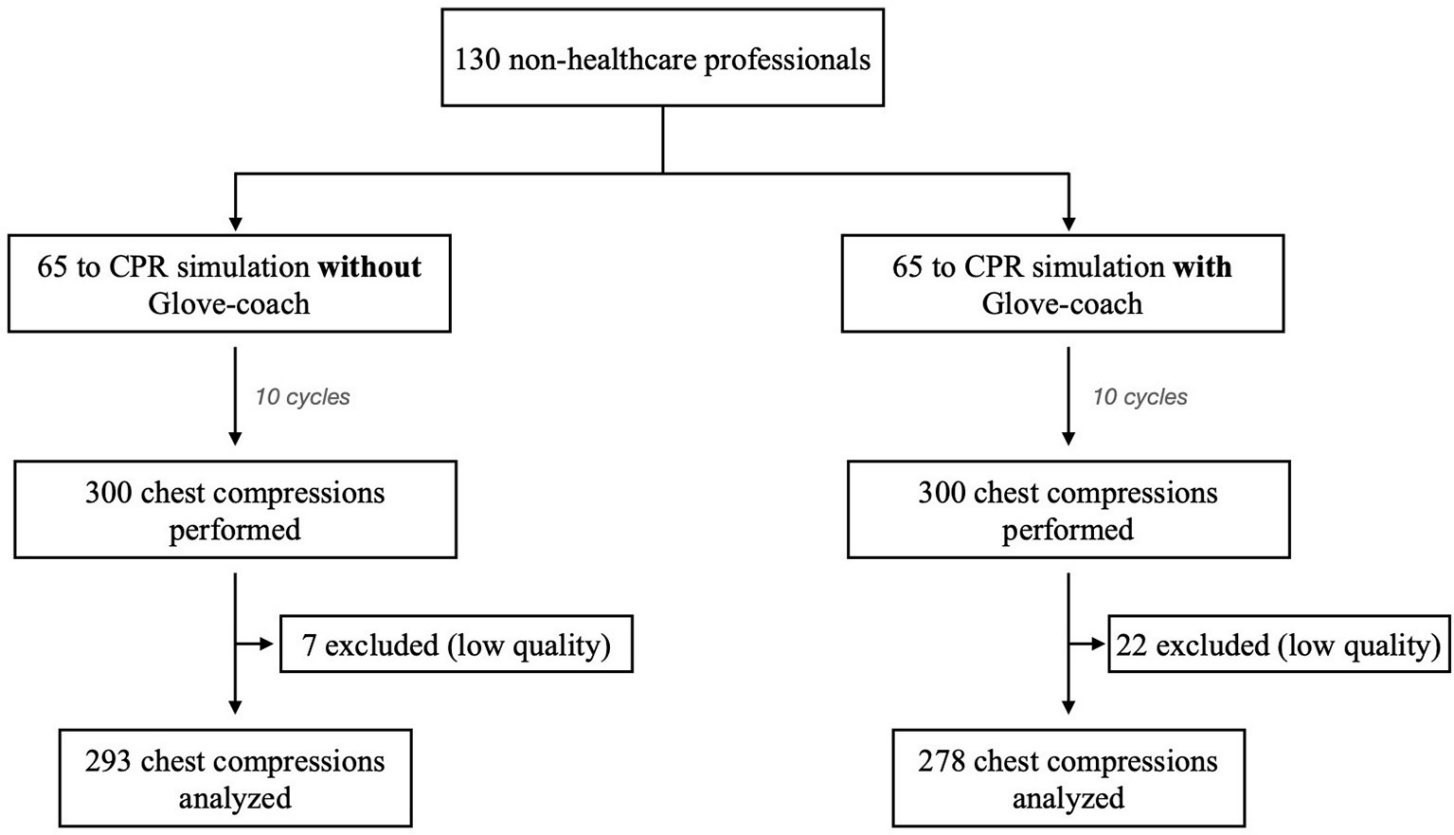
CPR data distribution: patients and chest compression distribution according to CONSORT standard.

One hundred and thirty volunteers were recruited and allocated to 1:1 ratio in both groups; mean age was 36 ± 15 years (min–max 21–64), and 62 (48%) were men; all volunteers performed some previous experiences in CPR procedure training, especially during school years or during voluntary activities. Starting from 65 volunteers for each group, a total of 600 chest compressions were performed, excluding chest compression that cannot be interpreted by the electromechanical model AmbuMan due to a low signal (22 in the *glove group*, seven in the *control group*), a total of 571 chest compressions were analyzed: 278 in the glove group and 293 in the control group (Figure 3 and Table 1).

**Table 1:** Patients' characteristics and chest compression data.

	**Control group**	**Glove group**	***p***
Healthy lay volunteers *(n)*	65	65	-
Age (years)	36 ± 14 (21 to 64)	37 ±15 (21 to 64)	-
Sex male (n)	32 (49%)	30 (46%)	-
CPR cycles *(n)*	10	10	-
Chest compressions analyzed *(n)*	293	278	-
Mean frequency *(rpm)*	103.02 ± 7.48 (100.1 to 158.37)	117.67 ± 18.63 (98.9 to 124.5)	<0.001
Percentage of cycle >100 rpm *(%)*	71 (35 to 100)	92.4 (87 to 100)	<0.001
Mean depth of compression *(mm)*	55.17 ± 9.09 (47.9–67.9)	52.11 ± 7.82 (42.7 to 65.3)	<0.001
Percentage of compression <5 cm *(%)*	18.1	26.4	0.004
Decay of compression depth *(mm/min)*	5.3 ± 1.28 (−3.5 to 7.7)	0.89 ± 2.91 (−3.3 to 4.1)	0.008

*Patients' characteristics and data distribution between control and glove group about depth and frequency of chest compression during CPR simulation in both groups. Rpm, rate per minute. When appropriate, data are expressed as mean ± SD (min–max)*.

Mean rate of compressions was 117.67 rpm ± 18.63 (min–max 98.9–124.5 rpm) in the glove group vs. 103.02 rpm ± 7.48 (min–max 100.1–158.37 rpm) in the control group (*p* < 0.001). Although the mean frequency of compression was significantly lower in the control group, in both groups, the compression rate was on average above the recommended threshold of 100 rpm. The percentage of compression cycles with an appropriate rate (>100 rpm) was 92.4% (min–max 87–100%) in the glove group vs. 71% (min–max 35–100%) in the control group, with an observed difference of 21.4% between the two groups, which resulted statistically significant (*p* < 0.001).

**Figure 4:**
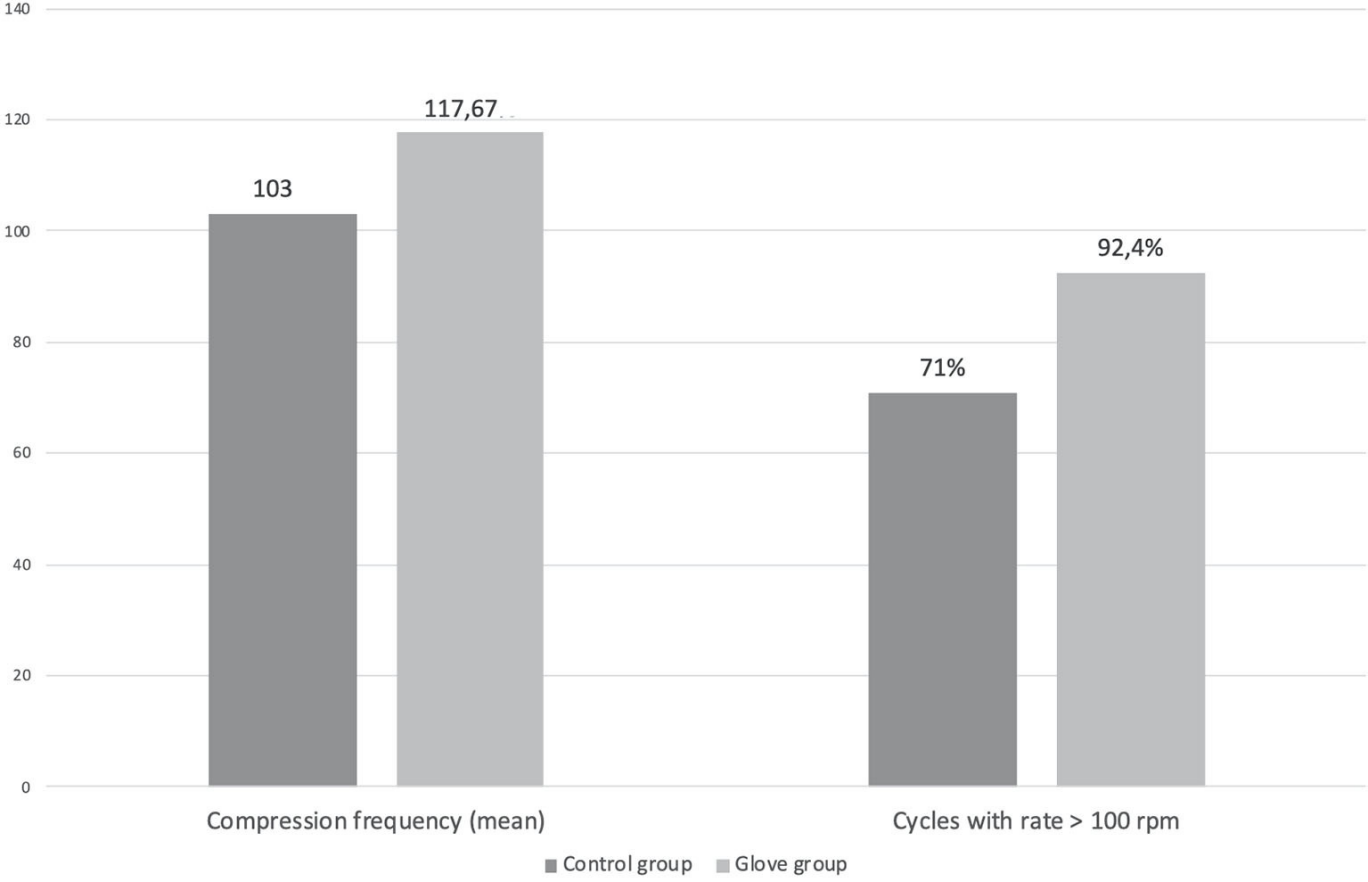
CPR depth and frequency distribution: differences about compression frequency and cycles with a rate >100 rpm during CPR between the two groups. All values are intended as mean values.

Mean compression depth was 52.11 mm ± 7.82 (min–max 42.7–65.3) in the glove group vs. 55.17 mm ± 9.09 (min–max 47.9–67.9) in the control group (*p* < 0.001). Although the depth of the compressions was significantly higher in the control group, in both groups, the mean compression depth was thus overall adequate as current recommendations (Figure 4). The difference in the percentages of compressions with an inappropriate depth (<5 cm) was statistically significant between the two groups (18.1% in the control group vs. 26.4% in the glove group, *p* = 0.004).

A mean decrease of compression depth over time of 5.3 mm/min ± 1.28 (min–max 3.5–7.7) was observed in the control group vs. a mean reduction of 0.83 mm/min ± 2.91 (min–max 3.3–4.1) in the glove group (*p* = 0.008). After only 10 mins of CPR (five cycles), compression depth in the control group resulted 49.87 mm ± 2.11 vs. 51.31 mm ± 2.4 in the glove group (*p* < 0.001).

## Discussion

During the last decade, an important improvement in the quality of wearable technology in healthcare applications has been performed; its superior ergonomic profile provides a clear advantage, especially for prolonged continuous monitoring and wireless transmission of vital parameters. Devices currently available on the market aiming at implementing the CPR quality have been conceived for professionals (21); moreover, they need to be connected to external monitors, they are part of defibrillators, or they have external components that should be applied to patients (22–26). Although laypersons are essential to improve the clinical outcomes of victims with sudden cardiac arrest, their capacity to perform an efficient massage, at the right rate and the right depth, is of uttermost importance (27). For this reason, feedback or prompt devices providing real-time cardiac compression depth, rate, chest recoil, and cardiac compression feedback were studied, underlying their role in ameliorating the quality of hands-only CPR performance by laypersons, or in social security activities or public services, such as police forces, public transports, sport societies, train stations, post offices, and banks (28). Available devices supporting laypersons in performing an efficient hands-only CPR could result laborious to use. Some of them provide visible feedback, which requires looking away from the patient to check the feedbacks, and the placement of devices on the patient (29) or on the operator's hand, involving a specific setup that could be difficult to reproduce in real life (23). Finally, some mobile devices improving CPR quality or assisting CPR might cause rescuers to delay life-saving measures (30). Compared to the available CPR devices for laypersons, the “glove-coach” methodology as a CPR monitoring coaching system results faster and is easier to use.

Following the AHA suggestions and their evolution during the last decade (3, 31), we implemented the use of this “glove-coach” methodology as a CPR monitoring coaching system, with a consequent reduction of more than 20% in the inappropriateness of frequency during CPR. In this context, acoustic and visual feedback provided by the device coaching system, such as the monitor on the glove, was useful in dictating the correct frequency of compressions to BLS non-healthcare providers, translating it into a significantly more accurate CPR. This aspect was already tested in healthcare providers, observing how devices providing real-time feedback and mobile devices containing a CPR app or software were relevant in the CPR quality improvement (32). On the other hand, the use of this “glove-coach” methodology did not significantly affect compression depth, which was overall appropriate in both groups. It is relevant to observe that compression depth of <5 cm occurred after only 10 CPR cycles in the control group, and this induces us to speculate an even higher decay over time, potentially leading to an ineffective CPR.

The frequency of chest compressions is a very important factor in maintaining a minimal cardiac output during CPR (3). Cardiopulmonary resuscitation guidelines clearly state that a frequency of at least 100 compressions per minute should be maintained constantly during resuscitation maneuvers. For many reasons (lack of experience, fatigue, etc.), however, this is often not consistently achieved, especially for laypersons, as shown by previous studies (5). We have shown that a wearable device providing visual and acoustic feedback may significantly improve the performance of cardiac massage, without interfering with the procedure and compression rate (23, 30): providers can in fact concentrate on patients and on the execution of CPR, without checking the monitor (as in other existing devices specifically conceived to assist cardiopulmonary resuscitation) or counting compressions. By providing real-time feedback through vocal commands, the glove continuously supports laypersons during the entire CPR.

Quite surprisingly, the use of the device did not translate into a significant difference regarding the average depth of compressions, which were adequate also in the large majority of the control group. This could be due to the largely diffused BLS courses in Switzerland, usually attended by a large portion of the population in different contexts, such as working environments, schools, driving license courses, and the army. It has been shown that an interactive CPR assist device is extremely useful in a population without a high degree of training (33). For this reason, the use of these devices by untrained people could lead to a clinical advantage in terms of CPR quality greater than the one recorded in this Swiss context.

Our trial included only 10 min of five cycles of CPR, and this factor could additionally explain the absence of significant difference in the two groups, with regard to compression depth. Depth of massage is in fact related to a mechanized gesture, which is highly reproducible without a significant thoughtful effort, by simply exploiting leverage between large body articulations. On the other side, the frequency depends more on the provider's active perception of time, and also on the physical status.

This trial presented some limitations. First, the study did not take place *in vivo* but on mechanical models, which raised the attention of lay rescuers about the quality of the massage. Second, the duration of the CPR tested was certainly shorter than a medium CPR. In spite of this, factors such as the quality of the massage and decay over time were already visible during this short test, and it can be therefore speculated that, with a potential longer duration of CPR time, this gap between the two groups could increase. This potential development, however, had not been measured. Third, we collected only relevant parameters concerning the CPR evaluation, such as rate, depth, and decay of compressions, considering them as the only relevant aspects in a CPR coaching system. Fourth, the glove, a device already patented, is currently in the testing phase and requires structural and software improvements. However, the purpose of the study was to validate the effectiveness of the CPR coaching system associated with the glove, while further tests will be needed to improve the glove's technical characteristics.

## Conclusion

A wearable device, such as a glove able to provide to laypersons real-time feedback through a coaching system evaluating rate, depth, and decay of compressions, could aid in the performance of an appropriate CPR maneuver in the context of cardiac arrest. Even if technical improvements and further studies are needed to confirm these promising results, our innovative approach could potentially improve the efficacy of CPR provided by laypersons, therefore improving the clinical outcomes of victims of sudden cardiac arrest.

## Data Availability Statement

The raw data supporting the conclusions of this article will be made available by the authors, without undue reservation.

## Ethics Statement

The Ethics Committee of Canton Ticino approved the protocol, without requiring informed consent as an electronic model was used.

## Author Contributions

MM, AS, SC, and LC: conceptualization and methodology. MM, SC, and AG: formal analysis. MM and SC: data curation. MM, SC, MB, and MI: writing and original draft. AS, SC, MB, XC, and TC: writing, reviewing, and editing. All authors contributed to the article and approved the submitted version.

## Conflict of Interest

MM is a manager who participated in the design and patenting of the glove. The remaining authors declare that the research was conducted in the absence of any commercial or financial relationships that could be construed as a potential conflict of interest.

## Publisher's Note

All claims expressed in this article are solely those of the authors and do not necessarily represent those of their affiliated organizations, or those of the publisher, the editors and the reviewers. Any product that may be evaluated in this article, or claim that may be made by its manufacturer, is not guaranteed or endorsed by the publisher.
