# Supplementary material for: Can a Glove-Coach Technology Significantly Increase the Efficacy of Cardiopulmonary Resuscitation on Non-healthcare Professionals? A Controlled Trial
Source: Front Cardiovasc Med. 2021 Dec 9;8:685988. doi: 10.3389/fcvm.2021.685988 (PMC8695546; doi:10.3389/fcvm.2021.685988)
Supplement: Supplementary file 1 [file Presentation_1.pdf]

Can A Glove-Coach Technology significantly increase the Efficacy of Cardiopulmonary Resuscitation on non-healthcare professionals? A controlled trial

Michele Musiari, Andrea Saporito, Samuele Ceruti, Maira Biggiogero, Martina Iattoni, Andrea Glotta, Laura Cantini, Xavier Capdevila, Tiziano Cassina

The cardio pulmonary resuscitation device (1) comprises a glove (2) and a process module (3) integrated in the glove (2) and equipped with an activation button (19). The glove (2) has a first portion (2a) for covering and integral protection of the palm, back and fingers of a hand, and a second portion (2b) for covering and integral protection of the wrist of the hand.

The process module (3) has a microcontroller (5) and connected to the microcontroller (5) a radio telecommunication module (6) equipped with relative antenna (6a) and a cellular mobile phone card (6b) (SIM card), an accelerometer (8), a loudspeaker (10), a microphone (11), a visible light emission module (16), and a satellite tracking module (9) equipped with relative antenna (9a). All these components of the process module (3) are housed in a container (18) arranged in correspondence with the second portion (2b) of the glove (2).

The container (18) has a rigid, watertight structure which, in addition to insulating, also mechanically protects the components housed inside it. In particular, the container (18) is positioned in correspondence with the side of the second portion (2b) of the glove (2) which in use covers the upper side of the wrist of the hand.

The components of the process module (3) are mounted directly on a printed circuit board (PCB), with the exception of the activation button 11 which, having to be easily operated, is supported directly by the container (18), and the loudspeaker, which is also directly supported from the container (18). The process module (3) is connected by means of special wiring 4a to a battery (4) for power supply

integrated in the glove (2) in correspondence with the side of the second portion (2b) of the glove (2) which in use covers the lower side of the wrist of the hand.

The glove (2) is made of an impermeable material to fluids and has a construction suitable for integrating the process module (3) and the battery (4): the container (18) and the battery (4) in particular are incorporated in the thickness wall of the glove (2) and, in the foreseen case in which the glove (2) has a construction that includes at least two layers of material, they are placed in special openable and resealable pockets obtained between the two layers of material.

The glove (2) preferably has a differentiated construction between the first portion (2a) and the second portion (2b) suitably reinforced with respect to the first portion (2a) for the integration of the process module (3) and the battery (4) . In particular, the first portion (2a) of the glove (2) at the limit can be made from a single layer of material. The container (18) as referred to above is advantageously arranged in a protected position outside the area of the back of the glove (2) on which the resuscitator must exert pressure with the other hand for the execution of the heart massage.

Ultimately, the components of the resuscitation device (1) housed in the container (18), both for the special location of the container (18) and also for the protective function performed by the container (18) itself, are free from the risk of damage that it may accidentally be caused by any organic fluids and above all by the energetic pressure action on the back of the glove (2) by the other hand of the resuscitator.

Advantageously, the loudspeaker (10) and the visible light emission module (16), in the pose naturally assumed by the arm and hand of the resuscitator during the execution of the cardiac massage, are arranged in such a position that their emissions are perceived directly without obstacles and therefore without distortion or attenuation by the resuscitator who must not even take his eyes and attention away from the action he is carrying out. The process module (3) is configured to automatically perform, once activated, the location of the operator's position through the satellite location module (9), and the

generation and transmission through the radio telecommunication module (6) and the mobile telephone card 6b of a telephone message for assistance request containing the indication of the position of the operator. The process module (3) is also configured to automatically perform, once activated, a comparison between actual measurement values carried out at least by the accelerometer (8) and reference measurement values and, on the basis of the comparison result, generate in real time through the loudspeaker (10) and the visible light emission module (16) a feedback for the depth of the heart massage and for the frequency of the heart massage.

The process module (3) also includes a memory module (12) also housed in the container (18) and connected to the microcontroller (5). Preferably the device (1) also comprises a camera (not shown).
The memory module (12), for example an SD memory card, is advantageously also used for environmental recording during rescue as well as for recording the monitoring data detected in terms of adherence to the cardio pulmonary resuscitation sequence, correct frequency of chest compressions external, correct depth of external chest compressions, adequacy of pauses by identifying those due to the sequence versus the arbitrary / incorrect ones.

If the device (1) is also equipped with a camera, the environmental recordings include audio and video data. The device (1) also includes, as shown, a pressure sensor (13) which cooperates with the accelerometer (8) to make a precise assessment of the chest compression depth. The pressure sensor (13) is connected via a signal cable 13a to the microcontroller (5).

In particular, the microcontroller (5), processing the signal received from the pressure sensor (13) is able to compensate the offset of the measurement obtained from the signal received from the accelerometer (8). The microcontroller (5) also processes the signal received from the pressure sensor (13) also to discriminate a false from a true accelerometer reading (8).

The device (1) can optionally also comprise electrocardiogram sensors (14) and a suitable interface 14a between the electrocardiogram sensors (14) and the microcontroller (5). In particular, and the electrocardiogram sensors (14) are connected via signal cables (14b) to the microcontroller (5).

The pressure sensor (13) and the electrocardiagram sensors (14) if provided, are arranged in correspondence with the side of the first portion (2a) of the glove (2) which in use covers the palm of the hand. Optionally, the device can also comprise other sensors, for example a temperature probe, an oximeter, motion and speed sensors, etc, and/or a display connected to the microcontroller (5) and mounted directly on the container (18).

Finally, the device may comprise one or more environmental sensors integrated in the glove (2), for example a carbon monoxide sensor connected to a corresponding emitter of an alarm signal. To process the feedback to the operator, the microcontroller (5) runs a calculation algorithm whose input data are provided as seen by the accelerometer (8) and by the pressure sensor (13). The feedback emitted by the speaker (10) consists of a voice message whose content is related to the result of the calculation algorithm.

The visible light emission module (16) can comprise a LED bar (16a) and in this case the feedback emitted by the visible light emission module (16) is formed by a coded and incremental sequence (red = too weak compression - green = correct compression - red = excessive compression) of LEDs (16a) on, whose number, progression and color are correlated to the result of the calculation algorithm. It must also be noted that all feedback information is immediately and naturally perceptible as it stimulates two distinct senses, sight and hearing, which can be received simultaneously and without hierarchy of precedence by the resuscitator as they are led to the cerebral cortex by two distinct sensory pathways.

The operation of the cardio pulmonary resuscitation device, with reference to figures (1), (6) and (7), is briefly as follows. The rescuer (20) puts on the glove (2) and activates the process module (3) by pressing the button (19).

The process module (3), as soon as it is activated, automatically generates and sends an alarm SMS message to the health emergency service (22) via the radio telecommunication module (6), the antenna (6a) and the telephone card (6b) and in parallel through the loudspeaker (10) starts the vocal tutorial indicated with A in figure (7) and essentially relating to a series of preliminary instructions for the recognition of the patient's cardio-circulatory arrest (ACC) conditions (21).

The SMS telephone message contains the telephone number of the caller (device) and, if available via satellite location, its geographical coordinates. The display of the telephone number of the device allows the emergency health service (EMS) (22) to call the device itself to which it responds, with an integrated hands-free “hands-free” system who is performing CPR. During the phone call, which does not imply the interruption of the CPR sequence, the operator of the Health Emergency System will have both confirmation of the patient's clinical condition and the address where to send help.

The telephone call from the health emergency service 22 (EMS) is received automatically by the device, ie without activating any key, via the radio telecommunication module (6), the antenna (6a) and the phone card (6b) and automatically stops the voice tutorial A.

Upon indication of the emergency health service operator, or by autonomous decision, by pressing the activation button (19), the process module (3) starts, via the loudspeaker (10), the voice tutorial B relating to the sequence of cardiopulmonary resuscitation (CPR), and at the same time starts the processing of the feedback for the guided correction of the massage according to a first tutorial (A). To further assist the rescuer's action, the microcontroller (5) activates a metronome function, generating through the loudspeaker (10) a succession of "beeps" at a constant pace to time the execution of the external chest compressions.

The volume of the “beeps” is automatically modulated when the vocal feedback is superimposed, which will always be predominant. The microcontroller (5) acquires, as mentioned, from the accelerometer (8) and from the pressure sensor (13) the measurement of depth and frequency of external chest

compressions and, depending on the comparison between the real measurement and the reference measurement, generates a specific feedback.

In particular, a voice message is generated by the loudspeaker (10) with the request to increase / decrease the compression rate if the measured compression frequency is respectively lower / higher than a reference value of 100 ± 10 compressions per minute.

At the same time, a red light is generated by the visible light emission module (16) if the measured compression depth is insufficient or excessive, and a green light if the measured compression depth is within the reference value. The number of green LEDs lit is greater the closer the measured compression depth value is to the correct reference value, and similarly the number of red LEDs lit is greater the further the measured compression value is from the correct value of reference.

In the event that the process module (3) after activation does not receive a telephone call from the emergency medical service (22) within a set time limit, it automatically interrupts tutorial A and starts the voice tutorial relating to the request to make a telephone call by other means, for example a personal mobile phone, a satellite telephone, a landline telephone, to the relevant health emergency service. In this case, tutorial C is started at the appropriate time by the rescuer as a result of pressing the button (19).

In conclusion, the process module (3) then activates a tutorial that guides the healthcare professional step by step to perform quality and effective CPR and an on-site correction of the behavior to be adopted. The process module (3) in fact provides guidance, monitoring and feed-back functions through the use of voice and / or visual prompts that guide the performer in the right CPR sequence, corrective voice and / or visual prompts on frequency and depth of external chest compressions. In this way the device (1) unifies the two main links of the survival chain through which both the call to the health emergency service and the early heart massage are performed in a simple, safe, effective way, being guided by a system of recognition and correction of the work carried out by the operator.

The cardio pulmonary resuscitation device can have various interfaces with the outside (e.g. Wifi, Bluetooth, Aux In, Aux Out, USB, Bridge 2, 4G, including special means of wireless connection to a central internet server capable of concentrate, manage and maintain all the data of the interventions and trace the position of the intervention itself.The data can be used both for statistical purposes as a basis for training the operator who uses the device, and as a remote assistance system.

The connection to the central server can take place in different operating modes: offline, hybrid, or online.
